# Epidemiology of familial multiple sclerosis in Iran: a national registry-based study

**DOI:** 10.1186/s12883-022-02609-1

**Published:** 2022-03-05

**Authors:** Zahra Salehi, Amir Almasi-Hashiani, Mohammad Ali Sahraian, Fereshteh Ashtari, Seyed Mohammad Baghbanian, Nazanin Razazian, Abdorreza Naser Moghadasi, Asghar Bayati, Amir Reza Azimi, Nahid Beladimoghadam, Mohammad Hossein Harirchian, Maryam Poursadeghfard, Samira Navardi, Reza Shirkoohi, Hora Heidari, Mehran Ghaffari, Sharareh Eskandarieh

**Affiliations:** 1 Department of Immunology, School of Medicine, Tehran University of Medical Sciences, Tehran, Iran; 2 Multiple Sclerosis Research Center, Neuroscience Institute, Tehran University of Medical Sciences, Sina Hospital, Hassan Abad Square, Tehran, Iran; 3 Department of Epidemiology, School of Health, Arak University of Medical Sciences, Arak, Iran; 4 Isfahan Neurosciences Research Center, Isfahan University of Medical Sciences, Isfahan, Iran; 5 Department of Neurology, Booalicina Hospital, Mazandaran University of Medical Sciences, Sari, Iran; 6 Department of Neurology, Medicine Faculty, Kermanshah University of Medical Sciences, Kermanshah, Iran; 7 Department of Neurology, Shahrekord University of Medical Sciences and Health Services, Shahrekord, Iran; 8 Department of Neurology, Shahid Beheshti University of Medical Sciences, Tehran, Iran; 9 Iranian Centre of Neurological Research, Neuroscience Institute, Tehran University of Medical Sciences, Tehran, Iran; 10 Clinical Neurology Research Center, Shiraz University of Medical Sciences, Shiraz, Iran; 11 Cancer Research Institute, Imam Khomeini Hospital Complex, Tehran University of Medical Sciences, Tehran, Iran

**Keywords:** Multiple sclerosis, Familial multiple sclerosis, Sporadic multiple sclerosis, Iran

## Abstract

**Background:**

Admittedly, little is known about the epidemiological signatures of familial multiple sclerosis (FMS) in different geographical regions of Iran.

**Objective:**

To determine the epidemiology and the risk of FMS incidence in several provinces of Iran with a different ethnic population including, Fars, Tehran, Isfahan (Persians), and Mazandaran (Mazanis), Kermanshah (Kurds), and Chaharmahal and Bakhtiari (Lors).

**Methods:**

This cross-sectional registry-based study was performed on nationwide MS registry of Iran (NMSRI) data collected from 2018 to 2021. This system, registers baseline characteristics, clinical presentations and symptoms, diagnostic and treatments at regional and national levels.

**Results:**

A total of 9200 patients including, 7003 (76.1%) female and 2197 (23.9%) male, were participated. About 19% of patients reported a family history of MS; the order from highest to lowest FMS prevalence was as follows: Fars (26.5%), Chaharmahal and Bakhtiari (21.1%), Tehran (20.5%), Isfahan (20.3%), Mazandaran (18.0%), and Kermanshah (12.5%). Of all FMS cases, 74.7% (1308 cases) were female and 25.3% (442 cases) were male. FMS occurrence was much more common in females than males (*P-*value *=* 0.001). Further, the mean age at onset was 30 years among FMS cases. A substantially higher probability of relapsing-remitting MS and secondary-progressive MS was found among FMS cases than sporadic MS (SMS) (*P*_value = 0.001). There was no significant difference in Expanded Disability Status Scale (EDSS) scores between FMS and SMS. The majority of FMS cases were observed among first-degree relatives, with the highest rate in siblings. There was a significant association between MS risk and positive familial history in both maternal and paternal aunt/uncle (*P*_value = 0.043 and *P*_value = 0.019, respectively). Multiple sclerosis occurrence among offspring of females was higher than males (*P*_value = 0.027).

**Conclusions:**

In summary, our findings imply a noteworthy upward trend of FMS in Iran, even more than the global prevalence, which suggests a unique Atlas of FMS prevalence in this multi-ethnic population. Despite the highest rate of FMS within Persian and Lor ethnicities, no statistically significant difference was observed among the provinces.

## Introduction

With an incrementing burden worldwide, neurological disorders are the leading cause of disability and the second leading cause of death. Based on the recent study by the global burden of disease (GBD), multiple sclerosis (MS) was indicated among disorders that are followed by an increasing pattern of mortality and disability-adjusted life-years (DALY) rates [1].

Multiple sclerosis, as the most common non-traumatic disorder of CNS, damages white and grey matter of the brain as well as the spinal cord, leading to demyelination and neurodegeneration and a wide spectrum of clinical manifestations [2]. The global prevalence of MS is estimated to be more than 2 million people and is approximately 2 to 3 times more common in women than men [3]. Although the age of MS onset is usually 20–40 years old, it has been also reported in children and people aged 50 years and older, known as the pediatric-onset of MS (POMS) and late-onset MS (LOMS), respectively [4, 5]. The first clinical presentation of MS is termed clinically isolated syndrome (CIS), which is typically followed by four clinical patterns including, relapsing-remitting MS (RRMS), secondary-progressive MS (SPMS), and primary-progressive MS (PPMS), and progressive-relapsing MS (PRMS) [2, 6].

Based on several studies, it is well known that MS results from dysregulation of immune response. Despite decades of effort, the main cause of systematic inflammation and autoimmunity observed in MS remains to be elucidated [7]. Numerous studies have pointed to the crucial role of a genetic component in MS susceptibility which endorses the aggregation of MS in families [8–10]. The global prevalence of familial MS (FMS) has been recently reported to be about 11.8% [11]. Given the impact of both genetic and environmental influences (incl. Geographical and cultural characteristics), the prevalence of FMS shows an uneven distribution among the different populations [5, 12]. Similar to sporadic MS (SMS), the age of onset in FMS patients is typically ranged between 20 and 30 years old [5, 11, 13]. About FMS prevalence between males and females, there are controversial results [14–16]. According to family-based studies, the incidence of the disease is higher among first- and second-degree relatives, respectively. Moreover, it has been reported that FMS cases are more likely to present RRMS and SPMS [17, 18].

In recent years, the prevalence and incidence of both SMS and FMS have been increased in Iran. With a higher intensity in the central part of the country, Iran is currently considered as an area with a high rate of MS [19, 20]. Recently, a positive history of FMS among the Iranian population was found to be 3.3–26.7% [21]. Specifically, the trend of FMS in Tehran, the capital city of Iran, has increased significantly over the past 18 years; from 5% in 2003 to 13.04% in 2018 [5]. Having the highest rate of different immigration, ethnic diversity, and socio-cultural conditions have made Tehran at the center of research priorities to evaluate FMS [5, 22] and just a few studies have been performed regarding the FMS prevalence in other provinces of Iran [14].

Here, we used nationwide MS registry of Iran (NMSRI) data to estimate not only the prevalence and demographic characteristics of FMS in Iran, even to provide more clues about the epidemiologic statistics of FMS in different geographical regions and ethnicities.

## Materials and methods

### Study population

The present cross-sectional registry-based study was conducted on clinically definite MS patients’ data recorded between December 4th, 2018, and January 1st, 2021 through the Nationwide MS registry of Iran (NMSRI). Several experts from 10 hospitals, 1 deputy of treatment, 7 MS societies, 20 governmental and private clinics filled the questionnaires and registered subjects’ data in NMSARI [23]. The target population of the present study comprises all age and sex groups of 9929 MS patients in six provinces of Iran including, Tehran, Isfahan, Fars, and Mazandaran, and Kermanshah, and Charmahal and Bakhtiyari.

### Nationwide MS registry of Iran

The NMSRI, with high reliability and validity, was launched in 2018. The registry has been set up to collect epidemiological features such as the prevalence, incidence, clinical presentations and symptoms, diagnostic and treatments, hospital course, and outcomes of MS by recording the patient data along with the annual follow-up. Completeness and maximum coverage of patients are the most important features of the registry system [23]. Multiple sclerosis diagnosis was confirmed for all cases by neurologists using the 2017 McDonald criteria [24].

All cases and their-associated epidemiologic features in NMSRI were gathered with the received reports from all neurological departments at Iranian hospitals, clinics, and the Iranian MS Society (IMSS). The registration of information was done by a neurologist or some trained registrants. All the gathered data was transferred to the main registration system daily. This is a dynamic and follow-up registration system and patients were followed up every 6 months. The NMSRI includes information about (1) patient identification data: national identification (ID) number, full name and family name, gender, age, date of birth, place of residence in the previous year, and date of visit, (2) Family history of MS: MS history in the first-, second-, and third-degree and other relatives, (3) Disease characteristics and course: date of diagnosis, types of MS, progress to secondary-progressive MS, Expanded Disability Status Scale (EDSS) score, (4) Medications: the name of all medications received by the patient during the last 3 years, dates of starting and stopping of each medication, and the reason for medication discontinuation. The age at disease onset was defined as the age at which patients present the first neurological symptoms associated with MS [23].

### Patients’ classification

Patients who were the only family member with MS were classified into SMS. Further, to be classified as a FMS case, the patients had to have at least one affected relative.

According to the classification of the International Pediatric MS Study Group (IPMSSG), cases with less than 18 years of age were identified as having POMS [25]. Those between 18 and 49.9 years old, and patients with 50 years old and over at disease onset were categorized as adult-onset MS patients (AOMS) and LOMS, respectively [26].

Relatives were divided into four categories: (1) first-degree relatives including mother, father, siblings, and offspring and spouse; (2) second-degree relatives including grandmother, grandfather, maternal aunt/uncle, and paternal aunt/uncle; (3) third-degree relatives including maternal and paternal cousins; and (4) other relatives [16].

Additionally, MS patients subdivided into two groups according to the degree of kinship between the affected members: (1) families in which MS-affected patients belong to the same generation (e.g. affected siblings and cousins of the same generation), and (2) families in which MS-affected patients belong to two or more generations (e.g. affected parents and offspring or affected siblings and affected uncles/aunts, etc.) [27].

### Statistical analysis

To describe the data, mean and, standard deviation or number (percentage) were used. Data analysis was performed using likelihood ratio chi-square, a two-sample test of proportions, independent t-test, and binary logistic regression. All tests were performed at a significance level of 0.05 using Stata software 13 (Stata Corp, College Station, TX, USA).

## Results

In the present study, the data of 9200 MS patients with median age (SD) of 30.6 ± 8.9, 7003 (76.1%) being female and 2197 (23.9%) being male, from 6 different provinces of Iran were analyzed. Of these, 1750 were FMS (19, 95% CI = 18.2–19.8%) and 7450 were SMS (81, 95% CI = 80.2–81.8%). The population consisted of 729 cases with unknown family history. The order from highest to lowest FMS prevalence was as follows: Fars (26.5%), Chaharmahal and Bakhtiari (21.1%), and Tehran (20.5%), Isfahan (20.3%), Mazandaran (18.0%), and Kermanshah (12.5%). The prevalence of familial and sporadic MS in each studies province is reported in Table 1.

**Table 1:** Prevalence of familial and sporadic MS cases in studied provinces

Variables		Tehran	Mazandaran	Chaharmahal and Bakhtiari	Kermanshah	Isfahan	Fars	Total
FMS (%)		679 (20.5)	306 (18.0)	81 (21.1)	182 (12.5)	417 (20.3)	85 (26.5)	1750 (19.0)
SMS (%)		2632 (79.5)	1372 (81.8)	303 (78.9)	1267 (87.4)	1640 (79.3)	236 (73.5)	7450 (81.0)
Age^a^ (S.D.)	FMS	30.2 (8.7)	30.5 (8.7)	30.8 (7.7)	32.2 (9.9)	31.1 (9.0)	29.2 (7.6)	30.7 (8.8)
	SMS	30.5 (9.0)	29.9 (8.6)	31.0 (7.4)	31.1 (9.4)	30.9 (9.3)	31.3 (8.4)	30.6 (9.1)
	***P*_value**	0.462	0.346	0.847	0.162	0.801	0.075	0.956
FMS (%)	Female	516 (76.0)	204 (66.7)	67 (82.7)	137 (75.3)	308 (73.9)	76 (89.4)	1308 (74.7)
	Male	163 (24.0)	102 (33.3)	14 (17.3)	45 (24.7)	109 (26.1)	9 (10.6)	442 (25.3)
	***P*_value**	**0.001**	**0.001**	**0.001**	**0.001**	**0.001**	**0.001**	**0.001**
FMS age (S.D.)	Female	30.2 (8.5)	30.3 (8.6)	31.2 (8.1)	32.7 (9.7)	30.8 (8.7)	29.3 (7.8)	30.6 (8.7)
	Male	30.1 (9.2)	30.9 (9.1)	28.8 (6.0)	31.0 (10.5)	32.2 (9.8)	28.2 (4.8)	30.8 (9.4)
	***P*_value**	0.861	0.606	0.287	0.338	0.216	0.752	0.819

^a^Age at disease onset

### Sex ratio

Of all FMS cases, 74.7% (1308 cases) were female and 25.3% (442 cases) were male. There was a significant difference in the prevalence of FMS between the two sexes (*P*_value = 0.001) and the results showed that the prevalence of FMS among women is significantly higher than men. The highest FMS prevalence was observed among females in Fars (89.4%). There was no remarkable difference between familial (female/male: 2.95) and sporadic cases (female/male: 3.24) in terms of sex ratio (Table 1).

### Age at disease onset

The mean age of disease onset was 30.66 years (SD = 8.99 years, 95% CI = 30.46–30.85). In details, the mean age of MS onset in FMS was 30.67 years (SD = 8.8 years, 95% CI = 30.22–31.11) and in SMS was 30.65 years (SD = 9.02 years, 95% CI = 30.43–30.87), respectively (Table 1). Overall, the results showed no significant difference in the age at disease onset between FMS and SMS (*P*_value = 0.956). Amongst FMS patients, the lowest and the highest age of disease onset was recorded in Fars (29.2 years) and Kermanshah (32.9 years), correspondingly. Notably, FMS patients exhibited an approximately earlier age of onset than SMS in Fars (29.2 vs. 31.1, *P*_value = 0.07). Moreover, there was no significant gender difference in age of disease onset among FMS patients (males = 30.8 and females = 30.6 years old, respectively (*P*_value = 0.819)) (Table 1).

In Table 2, the distribution of age groups is represented by familial or sporadic MS. The likelihood ratio chi-square revealed that there was no significant difference in the occurrence of familial or sporadic MS in different age groups. Among familial MS, 46.3% were 18–29 years old. Logistic regression revealed the odds of FMS in age groups as follows: 18–29 (OR: 0.98, 95% CI: 0.78–1.21), 30–39 (OR: 0.95, 95% CI: 0.75–1.21), and 40–49 (OR: 0.98, 95% CI: 0.75–1.29), and ≥ 50 (OR: 0.95, 95% CI: 0.62–1.45).

**Table 2:** The distribution of age groups at disease onset among familial and sporadic MS cases

Age Groups	SMS	FMS	Total	OR (95% CI)
< 18	442 (6.8)	101 (6.6)	543 (6.8)	**Reference**
18–29	3043 (46.9)	703 (46.3)	3746 (46.7)	0.98 (0.78–1.24)
30–39	2118 (32.6)	506 (33.4)	2624 (32.8)	0.95 (0.75–1.21)
40–49	740 (11.4)	171 (11.3)	911 (11.4)	0.98 (0.75–1.29)
≥50	150 (2.3)	36 (2.4)	186 (2.3)	0.95 (0.62–1.45)

### Multiple sclerosis types

As shown in Table 3, in both familial and sporadic cases, the most common form of the disease was RRMS (72.7%) which was followed by SPMS (15.7%). However, PRMS is the rarest form among cases (1.13). The likelihood ratio chi-square indicated a significantly higher probability of RRMS and SPMS among FMS cases (*P*_value = 0.001). There was not a significant difference in the mean EDSS scores between FMS and SMS groups (2.4 ± 1.9 vs. 2.3 ± 1.9, *P*_value = 0.122).

**Table 3:** The frequency of MS types among familial and sporadic cases

Type of MS n (%)	RRMS	PPMS	SPMS	PRMS	CIS
FMS	1026 (72.7)	60 (4.25)	22 (15.7)	16 (1.13)	87 (6.2)
SMS	4128 (70.42)	346 (5.9)	805 (13.7)	84 (1.43)	499 (8.5)
Total	5154 (70.86)	406 (5.6)	1027 (14.1)	100 (1.4)	586 (8.06)

*CIS* Clinically Isolated Syndrome, *RRMS* Relapsing-Remitting MS, *PPMS* Primary-Progressive MS, *PRMS* Progressive-Relapsing MS

### Family history

With an average of 1.26 (SD = 0.60) of the affected persons in studied families, 1246 (79.6%), 250 (15.96%), and 70 (4.53%) cases had one, two, and three or more persons with MS in their families, respectively. As shown in Table 4, there was no significant difference in the number of affected persons between males and females (*P*_value = 0.798).

**Table 4:** The number of MS patients among FMS cases

MS patients (n)	Female	Male	Total
1	948 (80.1)	298 (78.0)	1246 (79.6)
2	187 (15.8)	63 (16.5)	250 (15.96)
3	39 (3.3)	15 (3.9)	54 (3.5)
4	6 (0.5)	4 (1.05)	10 (0.64)
5	1 (0.08)	1 (0.26)	2 (0.13)
6	3 (0.25)	1 (0.26)	4 (0.26)

As shown in Table 5, among 1750 FMS patients, 1072 patients (61.3%) were in same generation and 429 patients (24.5%) were in different generations; since other relatives could not be categorized according to the generations affected by the disease, the sum of these two numbers is not 1. The frequency of MS patients among the same generation was significantly greater in women than men (62.7% vs. 57%, *P*_value = 0.035). However, the frequency of affected individuals among different-generation was not statistically significant between the two sexes (24.5 vs. 24.4, *P*_value = 0.964). The results showed that 28.6 and 24.6% of FMS cases had a history of disease in the maternal and paternal relatives, respectively. 2.6% of FMS patients had MS history in both paternal and maternal relatives. There was no significant difference among males and females regarding the history of disease in the maternal/paternal relatives (Table 5).

**Table 5:** Comparison of generation, maternal/ paternal, and pediatrics familial MS by gender

Variables	Female	Male	Total	***P***_value
**Same generation**	820 (62.7)	252 (57.0)	1072 (61.3)	**0.035**
**Different generation**	321 (24.5)	108 (24.4)	429 (24.5)	0.964
**Maternal**	373 (28.5)	127 (28.7)	500 (28.6)	0.931
**Paternal**	326 (24.9)	104 (23.5)	430 (24.6)	0.555
**Maternal/Paternal**	35 (2.7)	11 (2.5)	46 (2.6)	0.831
**Pediatric**^a^	70 (6.1)	31 (8.2)	101 (6.6)	0.174
**Adults**^b^	1069 (93.9)	347 (91.8)	1416 (93.4)	

^a^Cases with less than 18 years old at disease onset

^b^Cases with 18–50 years old and over at disease onset

Among FMS patients, 101 cases (6.7%) were pediatrics MS and 1416 cases (93.34%) were adults. The frequency of pediatric and adult FMS was not significantly different between females and males (Table 5).

The findings regarding the family relatives were as follows: 582 (38.9%) in first-degree relatives, 201 (13.1%) in second-degree relatives, 555 (36.1%) in third-degree relatives, and 199 (12.9%) were in other relatives. The most common family history was related to siblings (*n* = 538, 30.7%), followed by paternal cousin (*n* = 315, 18.0%).

Of the total cases, 543 cases (6.8%) were pediatric and 7467 (93.22%) were adults. The frequency of FMS among the pediatric affected population was similar to its frequency among adults and 101 cases of pediatric and 1416 cases of adult had the familial history (18.6% vs. 18.9%, *P*_value = 0.835). While there was a significant association between the history of MS among siblings (*P*_value = 0.047), a significant association was observed between the history of MS among fathers (*P*_value = 0.014), maternal aunt/uncles (*P*_value = 0.043), paternal aunt/uncle (*P*_value = 0.019) and pediatric MS (Table 6).

**Table 6:** Familial recurrence of MS regarding the degree of relatives

Relationship to proband	Pediatric MS	Adults MS	*P_value	Female	Male	*P_value
**First-degree relative**	35 (34.6)	566 (40.0)	0.291	523 (40.0)	168 (38.0)	0.462
Father	5 (4.9)	17 (1.2)	**0.014**	17 (1.3)	9 (2.1)	0.285
Mother	9 (8.9)	84 (5.9)	0.255	68 (5.2)	33 (7.5)	0.085
Sibling	22 (21.8)	438 (30.9)	**0.047**	412 (31.5)	126 (28.5)	0.236
Offspring	1 (0.99)	45 (3.2)	0.154	42 (3.2)	6 (1.4)	**0.027**
Spouse	1 (0.99)	23 (1.6)	0.598	13 (0.99)	16 (3.6)	**0.001**
**Second-degree relative**	25 (24.7)	199 (14.1)	**0.006**	196 (15.0)	60 (13.6)	0.465
Paternal grandmother/father	0 (0)	6 (0.42)	0.363	3 (0.23)	3 (0.68)	0.193
Maternal grandmother/father	0 (0)	11 (0.78)	0.217	7 (0.54)	5 (1.13)	0.214
Maternal aunt/uncle	14 (13.9)	109 (7.7)	**0.043**	102 (7.8)	36 (8.1)	0.816
Paternal aunt/uncle	12 (11.9)	78 (5.5)	**0.019**	88 (6.7)	19 (4.3)	0.056
**Third-degree relative**	29 (28.7)	472 (33.3)	0.335	446 (34.1)	135 (30.5)	0.168
Maternal cousin	10 (9.9)	239 (16.9)	0.052	228 (17.4)	65 (14.7)	0.180
Paternal cousin	21 (20.8)	254 (17.9)	0.479	238 (18.2)	77 (17.4)	0.713
**Others**	11 (10.9)	160 (11.3)	0.900	152 (11.6)	47 (10.6)	0.569

^*^Likelihood-Ratio Chi-Squared Test was used. The numbers in parentheses are “percentages” inside Table 6

### Familial MS transmission by sex

Table 6 presents a familial recurrence of MS according to the degree of relatives and sex. Among male MS patients, 3.6% of cases had a spouse with a history of MS, whereas, among females, it was less likely and the probability was estimated at 0.99% (*P*_value = 0.001). Multiple sclerosis occurrence among offspring of females was higher than males (3.2% vs. 1.4%, *P*_value = 0.027). In people with MS history in paternal aunt/uncle, FMS is more likely to occur in females than males (6.7% vs. 4.3%, *P*_value = 0.056) (Table 6).

## Discussion

To the best of our knowledge, the majority of the FMS studies in Iran have been performed in a cross-sectional setting which suffers from numerous limitations such as selection bias. Hence, the goal of the present study was to determine various aspects of FMS in Iran’s different ethnic groups through a registry system which made the current data more reliable than previous reports. All the information that was obtained from 9200 patients from six provinces with different ethnic populations including, Fars, Tehran, and Isfahan (Persians), Mazandaran (Mazanis), Kermanshah (Kurds), and Chaharmahal and Bakhtiari (Lors) were gathered through NMSRI during 2018 to 2021.

Overall, the result of this study showed that the prevalence of FMS in Iran was 19%. Hence, it is higher than the global prevalence estimated in recent studies (11.8% in 2021 and 12.6% in 2018) [11, 13]. A systematic review and meta-analysis study performed by Moosazadeh et al. in 2017 also reported a high rate of FMS among Iranian people, ranging from 3.3 to 26.7% [21]. While FMS in Iran was more prevalent than what was reported in Saudi Arabia [12], the estimated prevalence in our study was less than the prevalence reported in the study of Abu Dhabi by Ceccarelli et al. in 2020 [15]. In addition to being located in a region with a high prevalence of FMS, Iran has a growing trend of FMS which suggests a unique Atlas of FMS prevalence in this multi-ethnic population [20]. Given the complex nature of MS and the role of genetic contribution to MS risk, consanguineous marriage should be weighed among FMS patients in Iran, a country with high rates of parental consanguinity [28–30].

According to the subgroup analysis in terms of the province, the highest rate of FMS was in Persians and Lors, respectively. The results of a retrospective study on 871 MS patients in Shiraz between April 2004 and April 2018 described that 5.5% of patients had a history of MS in their family members [31]. Similarly, the current study and all our previous studies in Tehran indicated that FMS prevalence rose steadily; from 5% in 2003 to 13.04% in 2018 [22]. Nonetheless, no study was found that has evaluated FMS prevalence in Chaharmahal and Bakhtiari. In the current study, the prevalence of FMS in Isfahan [14, 32, 33] and Mazandaran [34, 35] was lower than in the previous. One study reported the 1.2% FMS prevalence in Kermanshah in 2012 [36]. Despite the higher rate of FMS within Persians, no statistically significant difference was observed among the provinces. It is important to note that the lowest FMS prevalence was found in Kermanshah and Mazandaran; they are located in the west and north of the country, respectively. As developed and industrial cities, a high frequency of FMS was found in Fars, Tehran, and Isfahan provinces. Strikingly, the discrepancies among the provinces highlight the existence of considerable variance in terms of both genetic and environmental factors such as the high stress of living in industrial cities, air pollution effects, and lifestyles across regions of a geographic area.

In the context of FMS gender distribution, FMS was found to be higher among Iranian females compared with males. Overall, this result is consistent with the results of earlier studies across other countries [17, 37]. Remarkably, females in Fars province showed the highest prevalence of FMS which might reflect the altered lifestyle factors within those females during the past decades. Making an explanation for the high prevalence of FMS among females in Fars could be an interesting topic for future studies.

In this study, the mean age of disease onset was 30.66 years. Furthermore, the mean age of patients at the time of disease onset was 30.7 and 30.6 years for FMS and SMS cases, respectively. The mean age of onset among Iranian FMS patients was more than 28.7 years which was reported in a worldwide study of 6114 FMS cases out of 15 studies [11]. However, the age of SMS onset was lower than 42.7 years reported in British Columbia MS (BCMS) database [38], 32.4 years reported in Argentina [39], and 31.33 years reported in Greece [40]. Almost identical results in MS age of onset were observed in Abu Dhabi and Italy [15, 38]. Concerning our findings, the youngest age of disease onset was found among FMS patients in Fars, which was estimated to be significantly lower in comparison to sporadic cases. Comparably, Ehtesham et al. reported the lowest age of disease onset in Shiraz city of Iran [11]. Given that Iran’s population growth rate is currently declining, the average age of MS incidence in Iran appears to be rising and approaching that of developed countries.

As regards the distribution of age groups, most of patients were identified as AOMS (91.6%) followed by POMS (6.8%) and LOMS (2.3%), respectively. The percentage of LOMS was approximately greater in FMS (2.4%) than SMS (2.3%). However, it should be noted that there was no significant difference in the mean age of onset neither between the two groups nor between the two genders within FMS patients. The present results are comparable to those reported by Mirmosayyeb et al.; of 2627 cases, 4.8% as early-onset of MS (EOMS), 3.20% as LOMS, and 91.93% as AOMS [26]. Further, 671 RRMS patients were evaluated in a retrospective long-term follow-up study. Of these, 143 (21.3%) were LORRMS (> 40 years) and 528 (78.7%) were young-onset RRMS (YORRMS; 18–40 years) [41].

Comparable to those results reported elsewhere [26], the most common form of disease in the present study was RRMS, which was followed by SPM, PPMS, and RPMS. Based on the data taken from the Isfahan Hakim MS database, LOMS patients had a higher risk of conversion from RRMS to SPMS in a shorter time (years) compared to EOMS and AOMS [26]. Although YORRMS showed more brain MRI inflammatory features, the survival curves analyses showed higher probability of reaching EDSS 6.0 for LORRMS in a shorter time (months) [41]. Remarkably, our results showed that FMS patients have a significantly higher rate of RRMS and SPMS types than SMS, although they show similar EDSS scores. Likewise, Steenhof et al. also described greater percentages of relapsing course in FMS compared to SMS [17]. The present findings showed the considerable impression of familial history on the disease course which should be taken in consideration.

The number of affected members is variable in family-based reports [5, 11]. In this study, the number of patients in a family ranged from one to six and the most of affected cases were in the same generation. The latter was significantly higher in women than men. Overall, MS risk was reported seven times more in patients with relatives having MS [42]. Like other studies [5, 16, 17], first- and third-degree relatives, especially siblings and paternal cousins of the affected individuals, were the prevalent kindship in the current study. Particularly, there was a significant association between already diagnosed adult MS patients and their siblings. Further, a significant association between pediatric MS and the affected father as well as both affected maternal and paternal aunt/uncles was identified. Previously, other studies also found a higher rate of MS development among parent-child relations [39, 43]. Particularly, 2–4% increased risk of childhood-onset of MS was evident in multi-incident MS families with a first-degree relative with MS (i.e. parent or sibling) [42, 44].

Based on our findings wives of MS probands experience an increased risk of MS compared to husbands. A nation world cohort study by Nielsen et al. found completely different results; i.e. “spouses of MS patients did not experience an increased risk of MS” and suggested no key role for environmental factors during adulthood [42]. We further assessed whether there was an association between paternal or maternal MS and the occurrence of disease in offspring. It was found that MS is more likely to be transmitted from mother to child than from father to child. Based on a similar study conducted by Steen Hof, it was recognized that MS is transmitted more from mother to child. Out of 133 cases of MS, 44 were between mother and child and 23 were between father and child [17]. Maternal illness during pregnancy, exposure to maternal passive smoking, and use of pesticides in the household during pregnancy may increase the chance the offspring will go on to develop MS.

### Limitation

The nationwide MS registry of Iran (NMSRI) has been held since 2018 and has not reached the maximum coverage yet. While Iran has thirty-one provinces, six of them have been examined in the present study. Hence, we might underestimate FMS prevalence in Iran. Since parents can be of different ethnicities, the exact ethnicity of the individuals is unknown. Further, measuring EDSS scores by different neurologists in different areas is associated with a considerable measurement bias.

## Data Availability

The data sets used and analyzed during the study are available from the corresponding author on reasonable request. All code will be provided by STATA software if required.

## Abbreviations

MS: Multiple sclerosis
FMS: Familial MS
SMS: Sporadic MS
CIS: Clinically Isolated Syndrome
RRMS: Relapsing-Remitting MS
SPMS: Secondary-Progressive MS
PPMS: Primary-Progressive MS
PRMS: Progressive-Relapsing MS
POMS: Pediatric onset of MS
LOMS: Late-onset MS
AOMS: Adult-onset of MS
EDSS: Expanded disability status scale
